# Ultra-sensitive detection of tumorigenic cellular impurities in human cell-processed therapeutic products by digital analysis of soft agar colony formation

**DOI:** 10.1038/srep17892

**Published:** 2015-12-08

**Authors:** Shinji Kusakawa, Satoshi Yasuda, Takuya Kuroda, Shin Kawamata, Yoji Sato

**Affiliations:** 1 Japan Agency for Medical Research and Development (AMED), Tokyo, Japan; 2 Division of Cell-Based Therapeutic Products, National Institute of Health Sciences, Tokyo, Japan; 3 Research and Development Center for Cell Therapy, Foundation for Biomedical Research and Innovation, Kobe, Japan; 4 Department of Quality Assurance Science for Pharmaceuticals, Graduate School of Pharmaceutical Sciences, Nagoya City University, Nagoya, Japan; 5 Department of Cellular and Gene Therapy Products, Graduate School of Pharmaceutical Sciences, Osaka University, Osaka, Japan; 6 Department of Translational Pharmaceutical Sciences, Graduate School of Pharmaceutical Sciences, Kyushu University, Fukuoka, Japan

## Abstract

Contamination with tumorigenic cellular impurities is one of the most pressing concerns for human cell-processed therapeutic products (hCTPs). The soft agar colony formation (SACF) assay, which is a well-known *in vitro* assay for the detection of malignant transformed cells, is applicable for the quality assessment of hCTPs. Here we established an image-based screening system for the SACF assay using a high-content cell analyzer termed the digital SACF assay. Dual fluorescence staining of formed colonies and the dissolution of soft agar led to accurate detection of transformed cells with the imaging cytometer. Partitioning a cell sample into multiple wells of culture plates enabled digital readout of the presence of colonies and elevated the sensitivity for their detection. In practice, the digital SACF assay detected impurity levels as low as 0.00001% of the hCTPs, i.e. only one HeLa cell contained in 10,000,000 human mesenchymal stem cells, within 30 days. The digital SACF assay saves time, is more sensitive than *in vivo* tumorigenicity tests, and would be useful for the quality control of hCTPs in the manufacturing process.

Human cell-processed therapeutic products (hCTPs) are eagerly expected to provide promising treatments for life-threatening and incurable diseases for which no adequate therapy is currently available. However, tumorigenic cellular impurities are a major concern for the manufacture and quality control of hCTPs transplanted into patients. Tumorigenic cells found in hCTPs as impurities are attributable to generation from the original component cells (e.g. spontaneous transformation) and/or cross-contamination with other tumorigenic cells. Human mesenchymal stem cells (hMSCs) are broadly used as hCTPs for the treatment of various diseases worldwide^1^,^2^, and they are believed to have little tumorigenic potential even after substantial manipulations of *in vitro* expansion^3^,^4^. As far as we know, four research papers have previously reported the *in vitro* spontaneous transformation of hMSCs^5^,^6^,^7^,^8^. Two of them, however, were retracted later because the cross-contamination of hMSCs with tumorigenic cells (fibrosarcoma, osteosarcoma, and glioma cell lines) was later identified as the cause of the results^9^,^10^. In the other two papers, the immortalization of hMSCs, which is closely associated with tumorigenicity, was initially observed in the *in vitro* culture, followed by confirmation with *in vivo* tumorigenicity tests^7^,^8^. These papers have shown two important points for the quality control of products derived from hMSCs in terms of tumorigenicity. First, to avoid cross-contamination, we should assess the contamination of hCTPs with tumorigenic cells and control the manufacturing processes. Second, *in vitro* monitoring of cell growth without senescence is quite useful for finding hCTPs containing immortalized cells^11^.

The soft agar colony formation (SACF) assay is a suitable method for monitoring anchorage-independent cell growth and is a well-known *in vitro* assay for the detection of malignant transformed cells^12^,^13^,^14^. In our previous study, the SACF assay was able to detect colonies generated from at least 0.1% HeLa cells spiked into hMSCs within 20 days^15^. We also suggested that its lower limit of detection (LLOD) of the assay signal means that it has the potential to detect hMSC contamination at approximately 0.02% HeLa cells. However, when the conventional SACF assay is applied to the process control in the manufacturing of hCTPs, much higher sensitivity of the assay for transformed cells would be needed to meet the quality assessment criteria of hCTPs. In practice, the cell numbers of hMSCs required are estimated at ~1 × 10^6^ cells/kg body weight and ~2 × 10^8^ cells/patient to treat graft-versus-host disease and ischemic heart disease, respectively^16^,^17^,^18^.

In the present study, we attempted to further develop an analyzing system for the SACF assay and established an image-based analyzing system that enables high-throughput screening of formed colonies. The goal of the present study was to demonstrate a feasible strategy for a highly sensitive SACF assay for the purpose of detecting transformed cells as tumorigenic impurities in hCTPs. Here we demonstrate a new analysis strategy termed “digital analysis” of the SACF assay.

## Results

### A single transformed cell spiked into hMSCs has the ability to form a colony in soft agar culture

**Figure 1:**
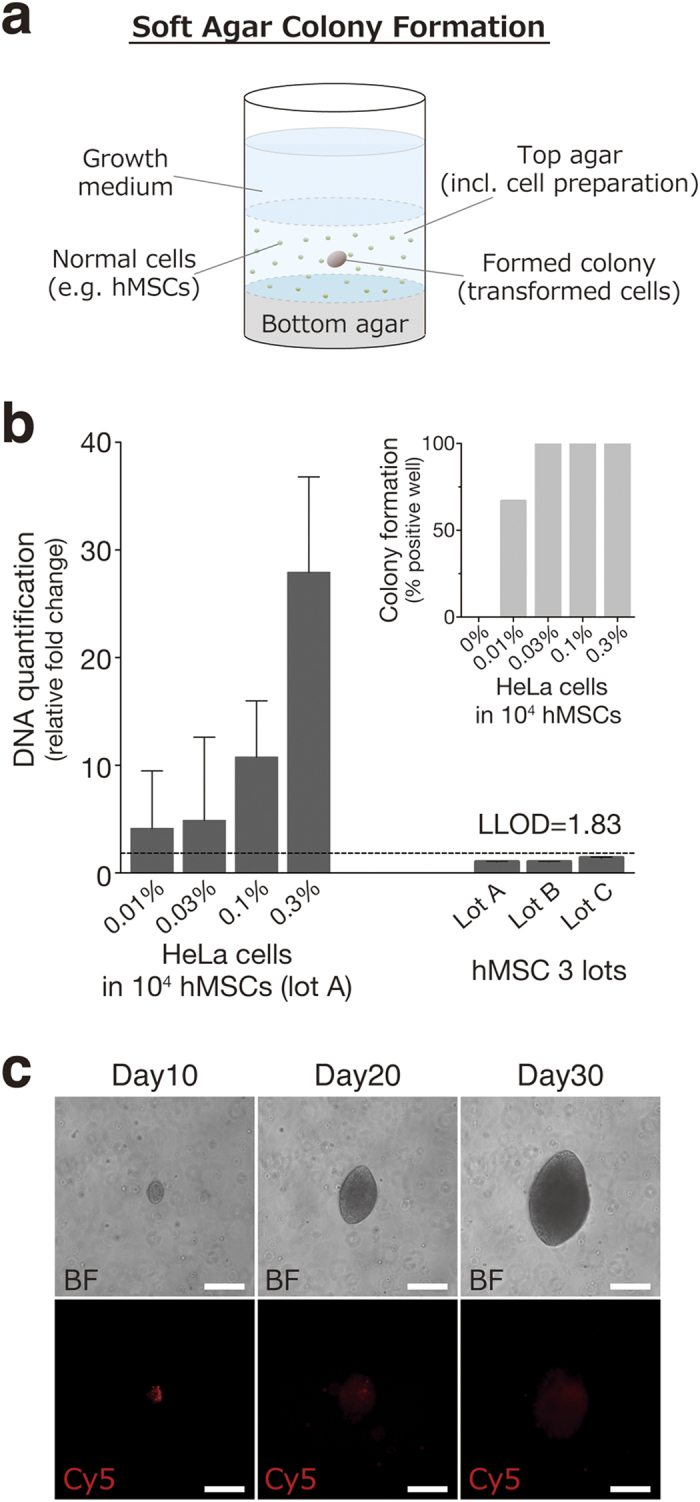
A single HeLa cell spiked into hMSCs has the ability to form a colony in soft agar culture. (**a**) A soft agar colony formation assay was applied for the detection of transformed cells contaminating non-tumorigenic human somatic cells. (**b**) HeLa cells (0%, 0 cells; 0.01%. 1 cell; 0.03%, 3 cells; 0.1%, 10 cells; 0.3%, 30 cells) spiked into hMSCs (10,000 cells) and three lots of hMSCs were grown in soft agar for 30 days. The LLOD was calculated as the mean plus 3.3 fold the SD of the measurement of the three lots of hMSCs. Quantification of the results is shown in (**b**). Cell growth was quantified using Cyquant GR dye. The results were expressed as the relative fold change of the value of the negative control (hMSC lot A). Error bars represent the SD of the measurements (n = 6). (**c**) Cell suspension containing HeLa cells labelled with CellVue and hMSCs were distributed into 48 wells of a 96-well culture plate at a concentration of one HeLa cell with 12,500 hMSCs per well and cultured in soft agar media for 30 days. CellVue-labelled cells were observed and tracked by fluorescence microscopic observation with a Cy5 filter for 30 days. Representative bright-field (BF) and fluorescent (Cy5) images of formed colony were shown (magnification, 100×; scale bars, 200 μm). hMSCs, human mesenchymal stem cells; LLOD, lower limit of detection.

In our previous study, the soft agar colony formation (SACF) assay (Fig. 1a) was applied for the detection of tumor cells contaminating non-tumorigenic human somatic cells as well as *in vivo* tumorigenicity tests. The SACF assay by quantification of cellular DNA detected colonies generated from at least 0.1% HeLa cells spiked into hMSCs within 20 days. The LLOD of the assay suggests that it has the potential to detect approximately 0.02% HeLa cells as impurities in hMSCs^15^. Here we first determined the actual LLOD of the SACF assay to detect HeLa cells contaminating the hMSCs. LLODs are commonly calculated as the mean + 3.3 × standard deviation (SD) of the background control^19^. We spiked several concentrations (0, 0.01, 0.03, 0.1 and 0.3%) of HeLa cells into 10,000 hMSCs and cultured them in soft agar media for 30 days to observe the minimum concentration of HeLa cells required for detection. The LLOD of the fluorescence assay for DNA quantification of the colonies was 1.83 based on the signals from three lots of hMSCs. The fluorescent signals detected from hMSCs containing 0.1% or more HeLa cells clearly exceeded the LLOD. On the other hand, the fluorescent signals of 0.01% and 0.03% HeLa cell-spiked hMSCs were variable around the LLOD, although they formed visible colonies (Fig. 1b). These results suggest that the DNA fluorescence assay of SACF is not able to detect 0.03% ( ≤ 3 cells) HeLa cells spiked into 10,000 hMSCs.

Next, we verified whether a single HeLa cell spiked into hMSCs actually form a colony in soft agar culture. We used HeLa cells labelled with CellVue, a fluorescent membrane dye, to identify a cell-of-origin for colonies formed in soft agar culture. The labelling efficiency of HeLa cells with CellVue was > 99% (Supplementary Fig. S1). Cell suspensions containing HeLa cells labelled with CellVue and hMSCs were distributed into 48 wells of a 96-well culture plate at a concentration of one HeLa cell with 12,500 hMSCs per well and cultured in soft agar media for 30 days. Visible single colonies expressing red fluorescence were observed within 10 days in 20 wells out of 48 wells, and these colonies grew in a time-dependent manner (Fig. 1c). These observations confirmed that a single HeLa cell spiked into hMSCs can form a single colony in soft agar culture. Other transformed cell lines, such as HT-1080, U87, and U251 cells, were also evaluated to assess their ability to form a colony from a single cell spiked into hMSCs in soft agar culture. Respective cell lines labelled with CellVue were distributed into 48 wells of a 96-well plate at a concentration of one transformed cell with 12,500 hMSCs per well. After 30 days of culture, colonies derived from a single transformed cell were observed for all three transformed cell lines (Supplementary Fig. S2). These results prompted us to measure the formation of a single colony derived from a single transformed cell in soft agar for the purpose of sensitively detecting tumorigenic cells as impurities in hCTPs.

### Image analysis of colonies derived from a single transformed cell

**Figure 2:**
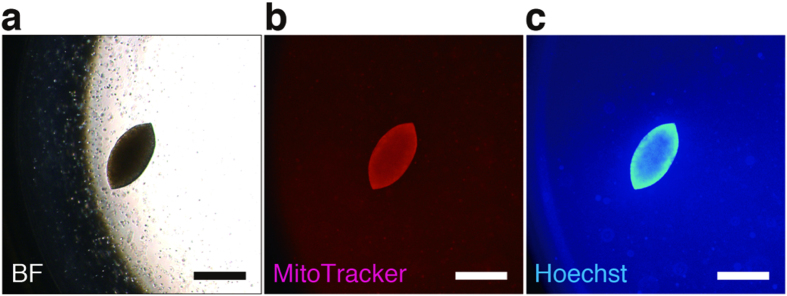
Fluorescent live cell staining of HeLa cell-derived colonies in soft agar culture. A single HeLa cell was co-cultured with 12,500 hMSCs per well of a 96-well plate in soft agar media. After 30 days of culture, cells were stained with MitoTracker Red CMXRos (final concentration = 25 nM) and Hoechst 33342 (final concentration = 1 μg/ml). Representative bright-field [BF (**a**)] and fluorescent [MitoTracker (**b)**; Hoechst (**c)**] images of the formed colony were shown (magnification, 100×; scale bars, 500 μm). hMSCs, human mesenchymal stem cells.

**Figure 3:**
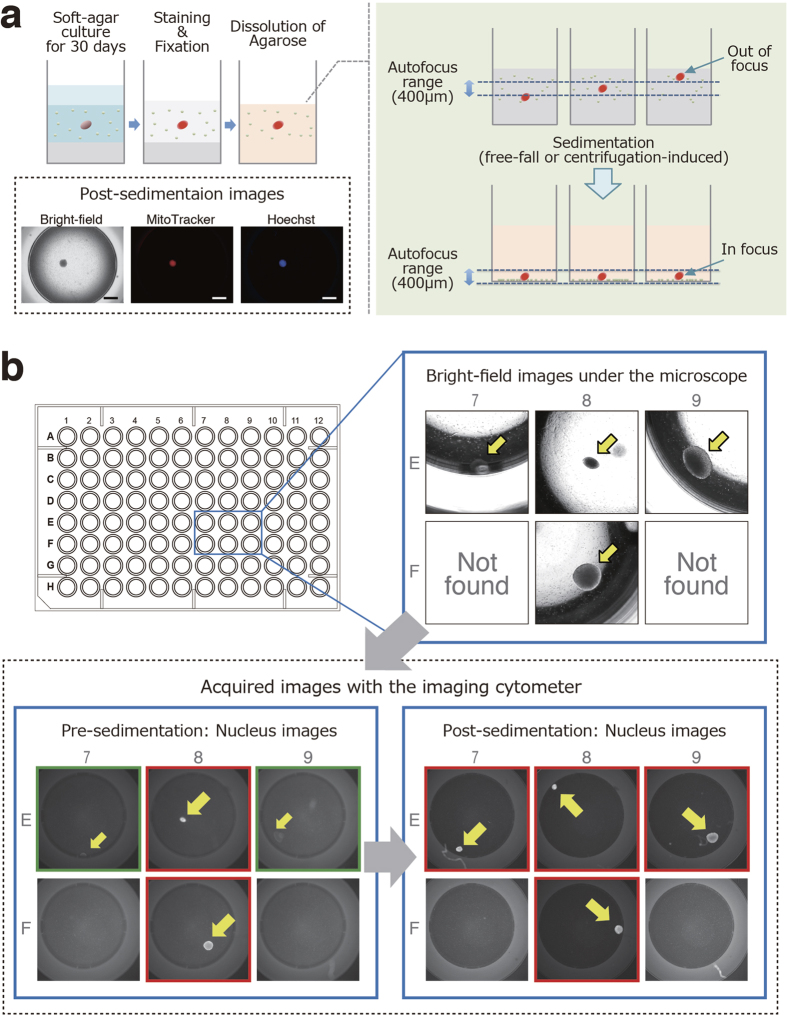
High-precision 2D imaging of colonies in soft agar culture using the imaging cytometer. (**a**) Although the imaging cytometer has the ability to autofocus on colonies formed in the soft agar media, it cannot actually recognize colonies out of the autofocus range (400 μm) of the equipped lens. To overcome difficulties in focusing on colonies in soft agar media with the imaging cytometer, the sedimentation of the floating colonies in the soft agar media into the bottom of the wells was attempted by dissolving the agarose. Representative post-sedimentation images are shown (magnification, 20×; scale bars, 1 mm). (**b**) Cell suspensions containing one HeLa cell and 12,500 hMSCs per well of 96-well plates were cultured in soft agar media for 30 days. Representative bright-field images of formed colonies with a light microscope are shown (upper right). These colonies were stained, fixed and subjected to high-content screening. Although some fluorescent images of colonies were not captured due to failed lens focus (green-edged images in the lower left panel), the sedimentation of the colonies enabled the imaging cytometer to clearly capture images of the colonies (red-edged images in the lower right panel). hMSCs, human mesenchymal stem cells.

We next attempted robust detection of a colony in a 96-well plate, with fluorescent dyes staining live cell colonies. Mitochondria and nuclei of live cells were stained with MitoTracker Red CMXRos and Hoechst 33342, respectively. MitoTracker Red CMXRos is a cell-permeable fluorescent dye [excitation wavelength (ex.) 579 nm; emission wavelength (em.) 599 nm] and selectively stains active mitochondria^20^. On the other hand, Hoechst 33342 is a cell-permeant blue fluorescent dye (ex. 350 nm; em. 461 nm) and is specific for DNA in the nucleus of live cells^21^. After incubation with 25 nM CMXRos and 1 μg/ml Hoechst 33342 for 1 h at 37 °C, the colonies of living cells clearly showed both red and blue fluorescence. Dual staining of the colonies with these dyes allowed us to visually distinguish a colony consisting of living cells from hMSCs in the background using fluorescence microscopy (Fig. 2). Since automated microscopy and image analysis systems have recently become available for high-content screening, we used an imaging cytometer, IN Cell Analyzer 2000, for further analysis of the SACF assay. Cell suspensions containing one HeLa cell and 12,500 hMSCs per well of 96-well plates were cultured in soft agar media. After 30 days of culture, various sizes of single colonies were observed in some of the wells with a light microscope (Fig. 3b, upper right). These samples were dually stained with fluorescent dyes, fixed with paraformaldehyde (PFA) and observed again with the imaging cytometer. However, all fluorescent images of colonies were not captured due to a failure of lens focus (Fig. 3b, lower left). Although the imaging cytometer has the ability to autofocus on colonies formed in the soft agar media, it cannot recognize colonies out of the autofocus range of the equipped lens. To overcome this difficulty in attaining proper focus on the colonies in the soft agar media when using the imaging cytometer, we attempted to sediment the floating colonies in the soft agar media at the bottom of the wells by dissolving the agarose. Buffer QG, a reagent provided by Qiagen that can dissolve agarose gel, successfully denaturalized the agarose without damaging the morphology or affecting the fluorescence intensity of the colonies (Fig. 3a). After the dissolution of the agarose, all of the colonies settled at the bottom of the wells within approximately 1 h due to gravity. We also confirmed that the sedimentation of the colonies was accelerated by centrifugation at 300 × *g* for 5 min without breaking the formed colonies (data not shown). The sedimentation of the colonies enabled the imaging cytometer to easily focus on the targets, thus allowing clear images of the colonies to be captured (Fig. 3b, lower right). In addition, colony sedimentation is likely to improve the laser-induced excitation efficiency for fluorescence and to contribute to high-precision imaging. Thus, combined application of our sample preparation and use of the imaging cytometer allowed us to acquire high-precision 2D images of colonies from each channel (red, blue, and bright-field) in 96-well plates.

We next scripted an image analysis that merged the different colors to detect transformed cell-derived positive colonies after the exclusion of the images of false-positive colonies (Supplementary Fig. S3). The areas showing fluorescence intensity were roughly segmented as an image at each color channel. The recognized areas were refined with a sieve post-processing operation. The criteria of filtering determined the target area at each color channel (size, > 6000; circularity, > 0.4; fluorescence intensity, > 100). Target areas overlapping at two channels were designated as positive colonies. We also checked these positive colonies by bright-field imaging to confirm the colony formation. Thus, we have established a cell image-based SACF assay using a high-content imaging system.

### Colony-forming efficiency of transformed cells in the soft agar culture

When spreading transformed cells at a quite low concentration onto culture plates, the number of wells containing transformed cells is based on the Poisson distribution^22^. If all of the transformed cells form colonies, the total number of colony-positive wells also follows the Poisson distribution. However, in practice, the transformed cells dispensed in each well do not always form colonies. Therefore, when establishing a colony-screening method, the colony formation efficiency of transformed cells needs to be shown as a positive control of the SACF assay. Cell suspensions were distributed into 80 wells of a 96-well plate at the predetermined concentration of HeLa cells, with 0, 12,500 or 62,500 hMSCs per well, followed by incubation in the soft agar media for 30 days. Then, the efficiencies of the single HeLa cell-derived colony formation were calculated, based on the number of colonies detected by the image analysis (Table 1). Colony image analysis revealed that fewer than half of the seeded single HeLa cells formed colonies (44.7 ± 2.2%). Colony formations of single HeLa cells co-cultured with hMSCs (12,500 and 62,500 cells) occurred at significantly higher frequencies (68.8 ± 9.2 and 70.0 ± 3.4%, respectively), showing the importance of the extracellular environment. Soft agar culture of hMSCs alone (12,500 and 62,500 cells) did not generate any colonies. The colony-forming efficiencies of the other transformed cell lines, HT-1080, U87, and U251, were also examined with or without 12,500 hMSCs per well (Supplementary Table S1). The colony-forming efficiency of U87 cells was comparable to that of HeLa cells. On the other hand, HT-1080 and U251 cells showed poor efficiency for colony formation. Consistent with the observations for HeLa cells, co-culture with hMSCs increased the colony-forming efficiencies of all of these transformed cell lines. In the SACF assay, the colony-forming efficiency of the positive control transformed cells (e.g. HeLa cells) enables us to estimate the number of transformed cells contained in the hCTPs, by assuming that they have the same colony-forming efficiency as the control.

### Digital analysis of SACF

**Figure 4:**
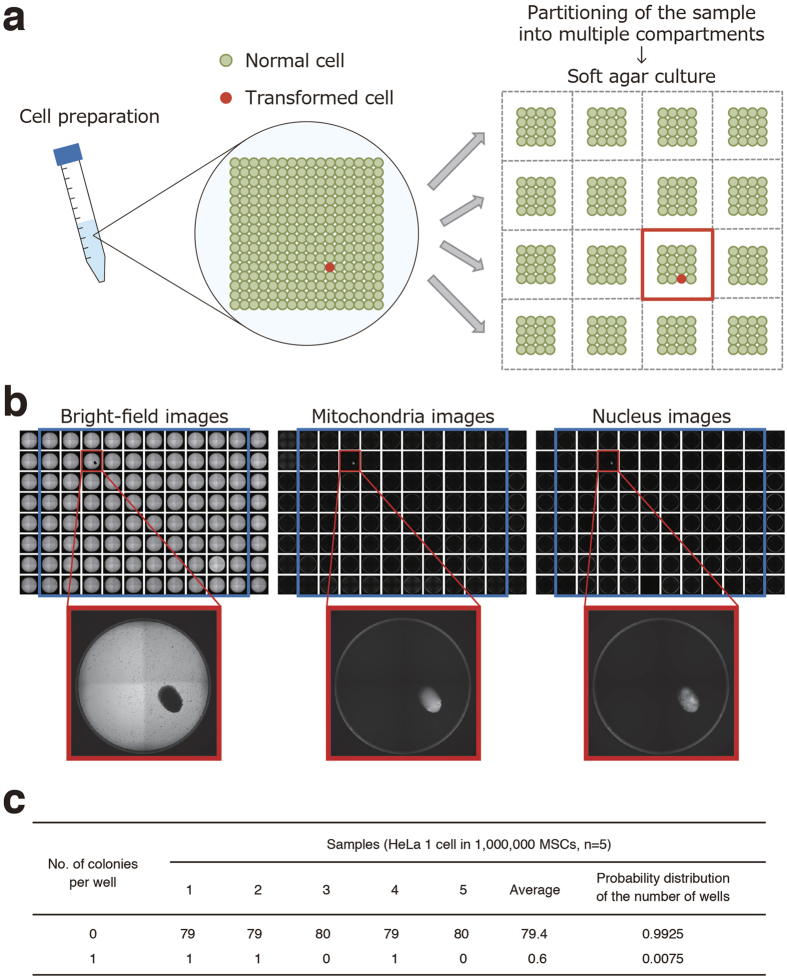
Digital analysis of soft agar colony formation for highly sensitive detection of a trace amount of transformed cells in hCTPs. (**a**) Schema of digital analysis strategy of soft agar colony formation. The strategy consists of partitioning the cell preparation into multiple compartments and then detecting whether colony formation has occurred or not in a compartment. When we equally divide a cell preparation into multiple wells of cell culture plates, so that each well contains 0 or 1 transformed cell, the wells should either be “positive” or “negative” for colony formation. (**b**) A single HeLa cell was picked up from a Terasaki microwell with a micropipette under microscopic visualization and mixed in a suspension of hMSCs (one million cells). When the cell suspension was aliquoted into 80 wells of 96-well plates at a concentration of 0.0125 HeLa cells with 12,500 hMSCs per well and cultured in the soft agar media for 30 days, our colony screening system successfully detected only one “positive” well, indicating its ability to detect concentrations of HeLa cells as low as 0.0001% in hMSCs. Representative bright-field and fluorescent images (mitochondria and nucleus) of one detected colony are shown. (**c**) The probability distribution values of the number of wells from the observed values of five experiments are shown in a table. hCTPs, human cell-processed therapeutic products; hMSCs, human mesenchymal stem cells.

**Figure 5:**
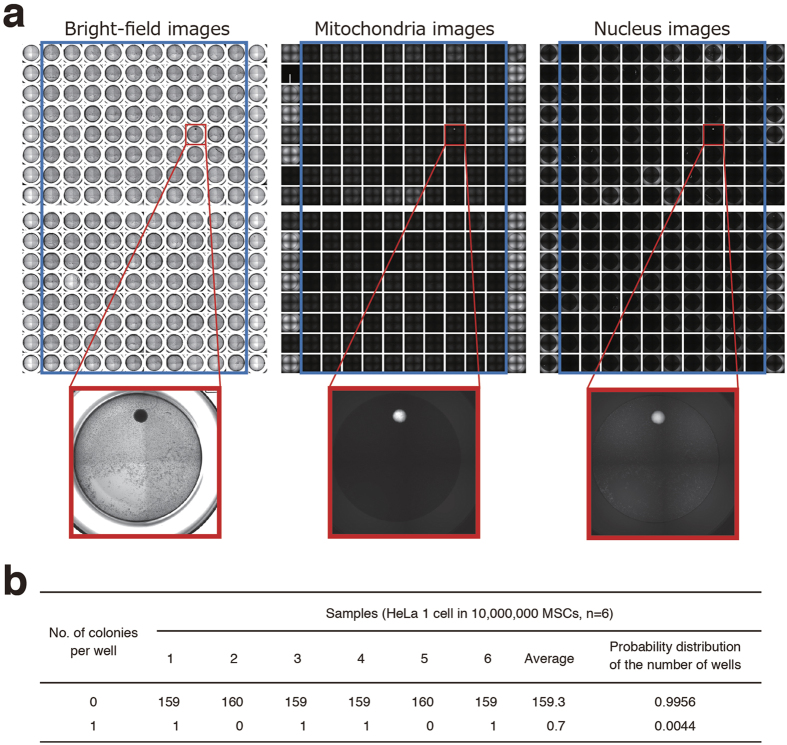
Digital analysis of soft agar colony formation can detect concentrations of HeLa cells as low as 0.00001% in hMSCs. (**a**) A single HeLa cell was picked up from a Terasaki microwell with a micropipette under microscopic visualization and mixed in a suspension of hMSCs (ten million cells). When the cell suspension was aliquoted into 160 wells of two 96-well plates at HeLa 0.0125 cells with 62,500 hMSCs per well and cultured in the soft agar media for 30 days, our colony screening system successfully detected only one “positive” well, indicating its ability to detect concentrations of HeLa cells as low as 0.00001% in hMSCs. Representative bright-field and fluorescent images (mitochondria and nucleus) of the detected colony are shown. (**b**) The probability distribution values of the number of wells from the observed values of six experiments are shown in a table. hMSCs, human mesenchymal stem cells.

We next tried to develop a new strategy for highly sensitive detection of a trace amount of transformed cells in the hCTPs using the high-content imaging system. We found that cell preparation for partitioning into multiple compartments is required when detecting only a small number of transformed cells (Fig. 4a). Partitioning of the cell sample was expected to dilute the background signal and to increase the signal-to-noise ratio of low-abundance transformed cells. To assess whether partitioning the sample elevates the sensitivity in detecting colonies of transformed cells, we spiked a single HeLa cell into 1,000,000 hMSCs (0.0001%) and split these cells into 80 wells of a 96-well plate for soft agar culture. Our imaging system successfully detected one well containing a single colony in three out of five experiments, indicating its ability to detect concentrations of HeLa cells as low as 0.0001% in hMSCs (Fig. 4b). Meanwhile, no colony formation was observed in five repeated experiments when only hMSCs were cultured in 80 wells of a 96-well plate with soft agar media. Thus, the division of the cell sample into multiple portions is suggested to be effective for detecting a small number of transformed cells in hCTPs by cell imaging. The probability distribution of the number of wells was 0.9925 and 0.0075 at 0 and 1 colony, respectively (Fig. 4c), and was used to estimate the false-negative rate of the whole assay (see the Discussion section). As each well contains only 0 or 1 transformed cell in a divided cell preparation, this is supposed to be indicative of “positive” or “negative” colony formation. Therefore, we named our novel strategy “the digital SACF assay” as its digital output data show either negative or positive. It is noteworthy that an increase in the partition number can theoretically make it sensitive for detecting transformed cells by the digital SACF assay. Next, we tested whether the digital SACF assay could detect only one colony derived from a single HeLa cell spiked into 10,000,000 hMSCs (0.00001%). The cells were distributed into 160 wells on 96-well plates at a density of 62,500 cells per well and cultured in the soft agar media for 30 days. The digital SACF assay successfully detected one positive well showing colony formation in four out of six experiments, whereas hMSCs alone did not result in the formation of any colonies in six control experiments (Fig. 5a). These results show that the digital SACF assay has the ability to detect HeLa cells at concentrations as low as 0.00001% in hMSCs. The probability distribution of the number of wells was 0.9956 and 0.0044 at 0 and 1 colony, respectively, when spreading cell samples consisting of one HeLa cell and 10,000,000 hMSCs into 160 wells of culture plates (Fig. 5b). The digital SACF assay can detect trace amounts of transformed cells in hCTPs, including large quantities of normal cells, with high sensitivity, owing to the number of partitions and divided culture cells.

## Discussion

The contamination of hCTPs with tumorigenic cells is an issue of concern for the manufacturing and quality control of hCTPs. The SACF assay is a traditional method to monitor anchorage-independent growth, which is one of the hallmark characteristics of cellular transformation and tumorigenicity^23^. Normal adherent cells will not grow in soft agar due to anoikis, a programmed cell death induced by cell detachment from extracellular matrix. On the other hand, transformed cells are able to grow and to form colonies in soft agar without anoikis. Therefore, colony formation is a specific phenomenon for tumorgenic cells. Cell tracking studies also confirmed that a single tumorigenic cell spiked into hMSCs formed a single colony in soft agar culture (Fig. 1c and Supplementary Fig. S2). Furthermore, soft agar culture of only hMSCs did not generate any colonies (Table 1). In the present study, we developed a new SACF method termed the digital SACF assay using a high-content imaging system to precisely detect the formation of a single colony derived from a single transformed cell in soft agar. The conventional DNA fluorescence assay of SACF is not able to detect 0.03% ( ≤ 3 cells) HeLa cells spiked into 10,000 hMSCs. On the other hand, our digital SACF assay detected a colony derived from a single HeLa cell in 10,000,000 hMSCs (0.00001%). Thus, our data suggest that the digital SACF assay is far superior to the conventional SACF assay by DNA quantification in terms of detection limit. When this assay shows colonies in a test product, some additional tests (e.g. short tandem repeat analysis) may provide further information about the origin of the colony forming cells.

Several imaging-based analyses of SACF with high-content imaging systems have recently been described^24^,^25^,^26^. We adopted the image analysis system for the SACF assay to directly identify the existence of a colony within a well. Some colonies in the soft agar can be overlooked depending on their height along the *z*-axis in a well due to the lack of lens focus (Fig. 3b); therefore, we dissolved the soft agar and aligned the colonies at the same height by the sedimentation of the floating cells (Fig. 3a). For the dissolution of the soft agar, we simply used an available reagent, Buffer QG, which can be used for DNA extraction from agarose gels. This reagent did not affect the morphology of the formed colonies and their fluorescence intensity with dyes. Here we combined MitoTracker and Hoechst 33342 dyes to dually stain colonies of live cells, excluding non-specific staining of debris with either of the two dyes. Dual staining of the colonies with these dyes allowed us to clearly distinguish live cells from dead cells. Because these fluorescences dominantly accumulate in colonies consisting of living transformed cells, we can also easily discriminate real colonies from debris or other artifacts as the background. These technical developments of SACF are critical for avoiding false-positive signals and conducting high-content image analysis.

In addition, we applied the concept of digital single-entity measurements^27^, including digital PCR^28^,^29^ and digital ELISA^30^, to the analysis of the SACF. The digital SACF assay was expected to increase the signal-to-noise ratio of low-abundance transformed cells due to the partitioning of the sample into multiple compartments (Fig. 4a). When a cell suspension containing one HeLa cell and 1,000,000 hMSCs (0.0001% HeLa cells) was aliquoted into 80 wells on a culture plate in the soft agar media, the digital SACF assay successfully detected only one “positive” well showing colony formation (Fig. 4c). Furthermore, when 10,000,000 hMSCs containing one HeLa cell (0.00001% HeLa cells) were distributed into 160 wells of culture plates, our digital SACF analysis successfully detected only one “positive” well (Fig. 5b). Thus, the digital SACF assay elevated the sensitivity for detecting the transformed cells grown in the soft agar by adjusting the number of cultured cells and partitioning them. In other words, according to the cell number of hCTPs used for clinical treatment, the digital SACF assay is able to adjust the content by the percentage of transformed cells for detection.

In the present study, we developed and characterized the digital SACF assay using HeLa cells, a well-known transformed cell line, as reference cells. Since the characteristics of the transformed cells as impurities in hCTPs cannot be predicted, we assumed that their colony-forming efficiency will be comparable to that of the reference cells in the digital SACF assay. With the use of positive control cells, we need to know the sensitivity of detection as well as the false-negative rate in the digital SACF assay for the quality evaluation of hCTPs. The false-negative rate of the digital SACF assay can be estimated from the proportion of partitions in which colony formation is not detected. We calculated the probability distribution of the number of wells showing no colonies from the data of the repeated experiments (Figs 4c and 5b). According to Fig. 4c, the false-negative rate of one well, i.e. the probability distribution of the number of wells showing no colonies, is 0.9925 for the detection of 0.0001% HeLa cells in hMSCs. The false-negative rate of one experiment using 80 wells (x) can be calculated as follows: x = 0.9925^80^ = 0.5476. The false-negative rate of a repeated number (n) of experiments (y) can be expressed as follows: y = x^n^. Hence, we obtain: n = log y/log x. When we set the permissible false-negative rate (y) at 0.01 (1%), the repeated number (n) of experiments can be calculated as follows: n = log (0.01)/log (0.5476) = 7.648. Therefore, eight experiments should be performed to rule out contamination with 0.0001% HeLa cells at a false-negative rate of less than 1%. Similarly, according to Fig. 5b, the false-negative rate of one well is 0.9956 for the detection of 0.00001% HeLa cells in hMSCs. The false-negative rate of one experiment using 160 wells (x) can be calculated as 0.9956^160^ = 0.4938. Permitting a false-negative rate of 1%, seven experiments are necessary to prove the absence of 0.00001% transformed cells comparable to HeLa cells [log (0.01)/log (0.4938) = 6.526].

We previously developed a highly sensitive *in vivo* tumorigenicity test using severely immunodeficient NOD/Shi-scid IL2Rγ^null^ (NOG) mice in combination with Matrigel^15^. When hMSCs (10^7^ cells) containing HeLa cells were subcutaneously injected into NOG mice with Matrigel, half of the mice were estimated to show tumor formation at a dose of 1.8 × 10^2^ HeLa cells (0.0018%) within 16 weeks. Although animal experiments are generally useful for confirming tumorigenicity of hCTPs, they are time consuming and costly to conduct. Our digital SACF assay saves time and is highly sensitive for the detection of HeLa cells compared with *in vivo* tumorigenicity tests. The digital SACF assay may compensate for the disadvantages of animal experiments by detecting trace amounts of transformed cells in hCTPs in the manufacturing process. To our knowledge, the digital analysis of SACF is one of the most sensitive methods to be reported for detecting trace amounts of transformed cells in hMSCs. We need to examine whether digital analysis of SACF can also sensitively detect transformed cells in the samples of other types of cells besides hMSCs to assess the versatility of our digital SACF assay in future. In that case, the choice of positive/negative control cells for the test cells may need to be adequately considered. Additionally, further technical optimization of the digital SACF assay may be helpful not only for increasing its sensitivity and specificity for the detection of cellular impurities in hCTPs, but also for ensuring the safety and quality of hCTPs.

## Methods

### Cells

Human cervical cancer cell line HeLa, human sarcoma cell line HT-1080, and human glioblastoma cell lines U87 and U251 were obtained from the Health Science Research Resources Bank (Osaka, Japan). These cell lines were maintained in Eagle’s minimum essential medium (MEM; Sigma, St. Louis, MO, USA) supplemented with 10% fetal bovine serum (FBS; Sigma), 100 U/ml penicillin and 100 μg/ml streptomycin (Nacalai Tesque, Kyoto, Japan). hMSCs were purchased from Lonza and cultured in MSCGM^TM^ medium (Lonza, Basel, Switzerland). Cells were cultured in a humidified atmosphere of 5% CO_2_ and 95% air at 37 °C and were passaged upon reaching 80% confluence.

### Soft agar colony formation assay

Black wall glass-bottom 96-well plates (Greiner, Kremsmünster, Austria) or clear, flat-bottom non-treated 96-well plates (BD Falcon, Franklin Lakes, NJ, USA) were used in the SACF assay. Prewarmed 25 μl of 2 × Dulbecco’s modified Eagle’s medium (D-MEM; prepared from D-MEM powder; Wako, Osaka, Japan) containing 20% FBS, 200 U/ml penicillin, 200 μg/ml streptomycin and 25 μl of melted 1.2% SeaPlaque Agarose (Lonza) solution were mixed and transferred into a well in a 96-well plate, which was then incubated at 4 °C for 30 min to allow the bottom agar layer to solidify. Cells were dissociated into a single-cell suspension by treatment with 0.25% trypsin- EDTA solution (Life Technologies, Carlsbad, CA, USA) and passed through 40 μm nylon cell strainers (BD Falcon). Next, 25 μl of cell suspension in D-MEM/10% FBS was mixed with 25 μl of 2 × D-MEM containing 20% FBS and 25 μl of 1.2% agar. After being placed on the bottom agar layer, the top agar layers were immediately solidified at 4 °C for 15 min to avoid gravity-induced anchorage-dependent cell proliferation at the bottom of the wells. The plates were incubated with 100 μl of D-MEM containing 10% FBS per well for 30 days at 37 °C and 5% CO_2_. The medium was changed every 3–4 days. This soft agar culture method was performed under the same condition in all experiments, irrespective of colony screening methods.

### Fluorescence assay for DNA quantification of soft agar colonies

The conventional SACF assay was performed with the Cytoselect Cell Transformation Assay Kit (Cell Biolabs, San Diego, CA, USA) in accordance with the manufacturer’s instructions and our previous study^15^,^31^. Briefly, serially diluted 0, 1, 3, 10 and 30 HeLa cells and 10,000 hMSCs were suspended in D-MEM containing 10% FBS with 0.4% agarose and layered on the bottom agar layer in each well on 96-well plates. Cultures were maintained for 30 days at 37 °C and 5% CO_2_. Colony formation was determined using a light microscope and was measured by agar solubilization, followed by cell lysis and quantification of the cell number with CyQuant GR dye (included in the kit) in a fluorescence plate reader. The results were evaluated as the relative fold change of the value of the negative control (hMSCs only). The LLOD of the assay signal was calculated as the mean plus 3.3-fold the SD of the measurement of the three lots of hMSCs^15^.

### Cell tracking study in SACF

We used the fluorescent membrane dye CellVue (Sigma) to identify a cell-of-origin for the colonies formed in the soft agar media. Cell suspensions containing single transformed cells, which were labelled with CellVue according to the manufacturer’s instructions, and hMSCs were distributed into 48 wells of a 96-well culture plate at a concentration of one transformed cell with 12,500 hMSCs per well and cultured in soft agar media for 30 days. CellVue-labelled cells were tracked by fluorescence microscopy with a Cyanine 5 (Cy5) filter (All-in-One Fluorescence Microscope BZ-X700; Keyence, Osaka, Japan).

### Image analysis of SACF

After 30 days of SACF culture, media was removed from each well, and 25 μl of media containing 150 ng Hoechst 33342 (Dojindo, Kumamoto, Japan; final concentration = 1 μg/ml) and 150 nM MitoTracker Red CMXRos (Life Technologies; final concentration = 25 nM) were added to each well. Plates were incubated at 37 °C and 5% CO_2_ for 1 hour before fixation by adding 125 μl of 4% PFA in PBS to reach a final PFA concentration of 2% at room temperature for 30  min. Then, the PFA solutions were removed, the cells were washed twice with PBS and 50 μl of Buffer QG (Qiagen, Venlo, Netherlands) was added to denaturalize the agar media. Plates were incubated at 37 °C for a minimum of 1  h and stored at 4 °C until image analysis.

Assay plates were imaged with the imaging cytometer IN Cell Analyzer 2000 (GE Healthcare, Little Chalfont, UK). Imaging was performed to acquire four fields of view for each of the three channels (red, blue and bright-field) per well of a 96-well plate using a 4× objective lens. Four images from separate fields were stitched together to obtain an image of the whole well. Automated image analysis was performed on each well with IN Cell Developer Toolbox v1.9 software (GE Healthcare), utilizing the analyzing script to segment colony features based on size, circularity and fluorescence intensity-related criteria.

### Estimation of the colony-forming efficiency of transformed cells

The colony-forming efficiency of the transformed cells in the soft agar culture in one well of a 96-well plate was defined as the ratio of the total number of colonies to the number of transformed cells dispensed in a 96-well plate. The cell suspension was prepared via a dilution series from the cell preparation at around 100 cells/ml, which was estimated from the sum of manually counted cell numbers in each well under microscopic visualization when 10 μl of the cell preparation was dispensed into each of 40 wells on a Terasaki plate (Watson Biolab, Kobe, Japan). When the cell suspension at a concentration of 0.5 cells per well is distributed into 80 wells of 96-well plates, the total number of dispensed transformed cells is estimated to be 40. The cell suspension was distributed into 80 wells of a 96-well plate at the predetermined concentration (around 0.5 cells) of HeLa cells with 0, 12,500 and 62,500 hMSCs per well, followed by incubation for 30 days. The colony number in the 96-well plates was then counted by image analysis, and the colony-forming efficiency of the transformed cells was calculated.

## Additional Information

**How to cite this article**: Kusakawa, S. *et al.* Ultra-sensitive detection of tumorigenic cellular impurities in human cell-processed therapeutic products by digital analysis of soft agar colony formation. *Sci. Rep.* **5**, 17892; doi: 10.1038/srep17892 (2015).

## Supplementary Material

Supplementary Information

## Figures and Tables

**Table 1:** Efficiency of single cell-derived colony formation from a HeLa cell in soft agar culture with or without hMSCs (12,500 and 62,500 cells).

	HeLa	HeLa/hMSCs (12,500)	HeLa/hMSCs (62,500)	hMSCs (12,500)	hMSCs (62,500)
Efficiency of colony formation (%)	44.7 ± 2.2^a^	68.8 ± 9.2^#^	70.0 ± 3.4^#^	Not detected	Not detected

^a^The ratio of the total number of colonies to the number of transformed cells dispensed in a 96-well plate were defined as the colony-forming efficiency of transformed cells in soft agar culture. Values are means ± SD of three experiments. Statistical significance was determined using one-way ANOVA and Dunnett’s multiple comparisons test (^#^*P* < 0.05, compared with the HeLa cell alone group). hMSCs, human mesenchymal stem cells.
