# Supplementary material for: Ultra-sensitive detection of tumorigenic cellular impurities in human cell-processed therapeutic products by digital analysis of soft agar colony formation
Source: Sci Rep. 2015 Dec 8;5:17892. doi: 10.1038/srep17892 (PMC4672267; doi:10.1038/srep17892)
Supplement: Supplementary Information [file srep17892-s1.pdf]

Supplementary Information

Ultra-sensitive detection of tumorigenic cellular impurities
in human cell-processed therapeutic products
with digital analysis of soft agar colony formation

Shinji Kusakawa, Satoshi Yasuda, Takuya Kuroda, Shin Kawamata & Yoji Sato

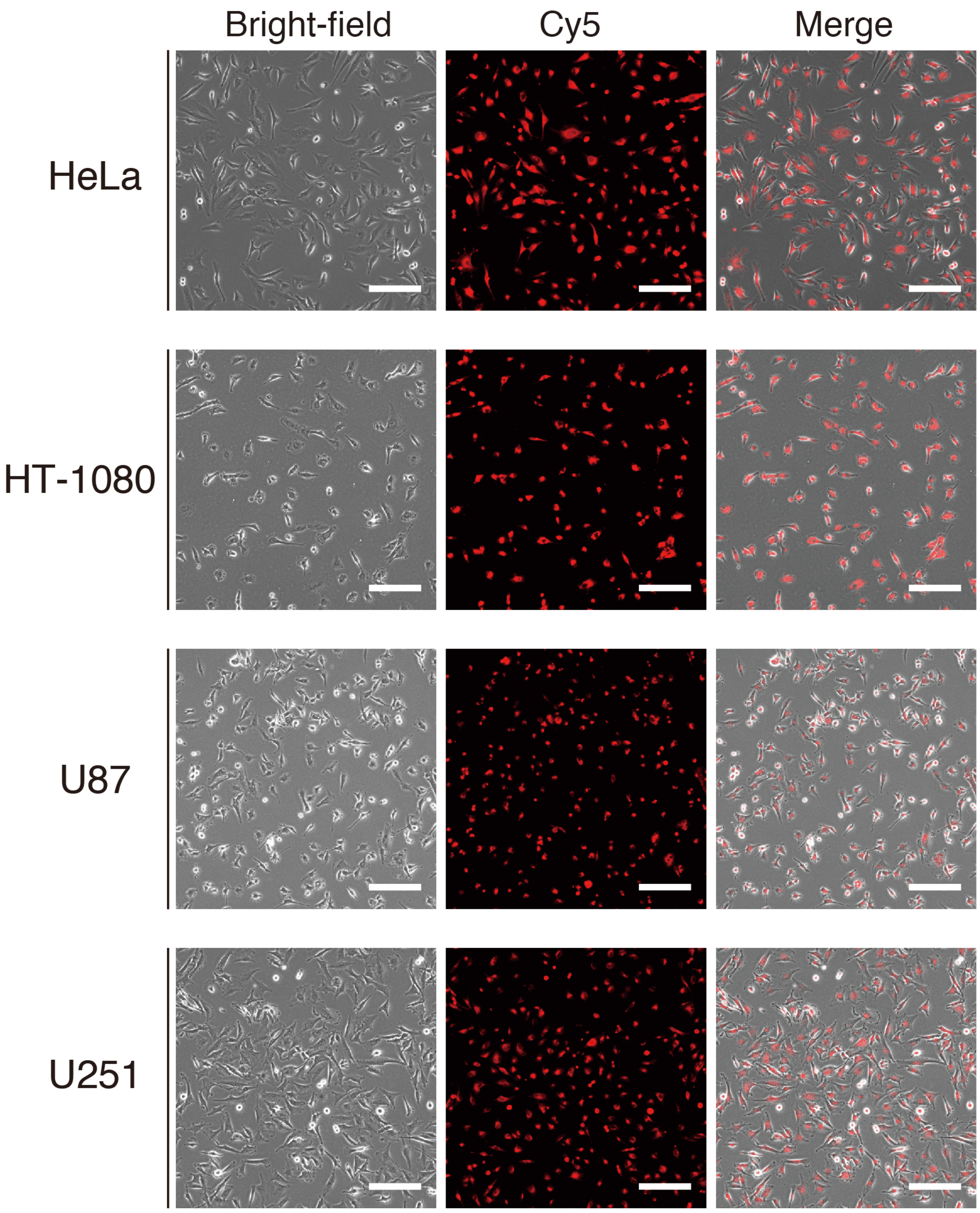

Figure S1. Fluorescent labelling of transformed cells with CellVue

Transformed cell lines (HeLa, HT-1080, U87, and U251 cells) were labelled with CellVue. After 24 h, CellVue-positive cells were observed by fluorescence microscopic observation with a Cy5 filter. Representative bright-field, fluorescent (Cy5), and merged images of transformed cell lines were shown (100x ; scale bars, 200 μm).

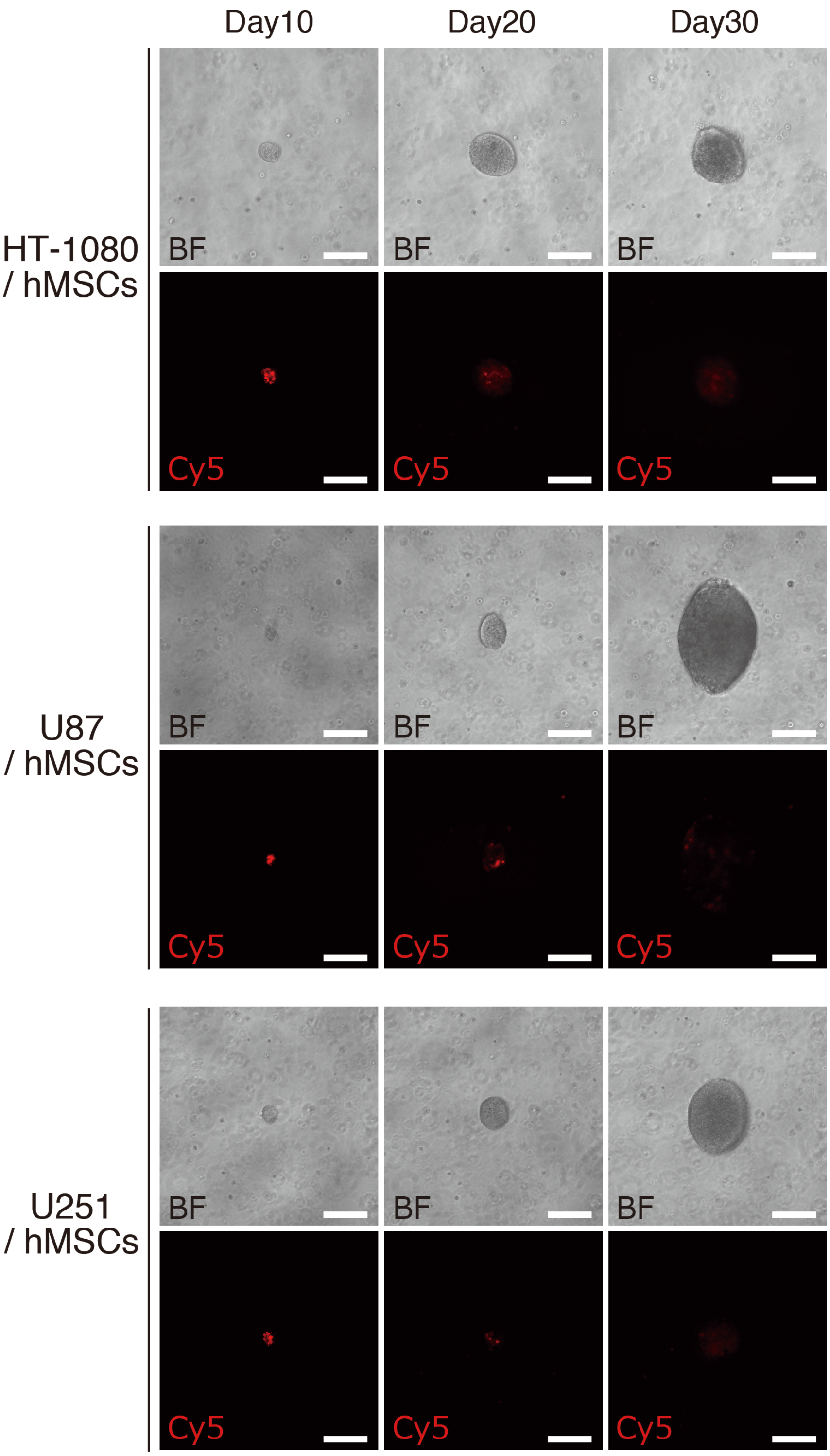

Figure S2. Various single transformed cells spiked into normal cells have the ability to form colonies in soft agar culture

Cell suspensions containing U87, U251 and HT-1080 cells labelled with CellVue and hMSCs were distributed into 48 wells of a 96-well culture plate at a concentration of one HeLa cell with 12,500 hMSCs per well and cultured in soft agar media for 30 days. CellVue-labelled cells were observed and tracked by fluorescence microscopy with a Cy5 filter for 30 days. Representative bright-field (BF) and fluorescent (Cy5) images of formed colony were shown (magnification, 100x ; scale bars, 200 μm). hMSC, human mesenchymal stem cells.

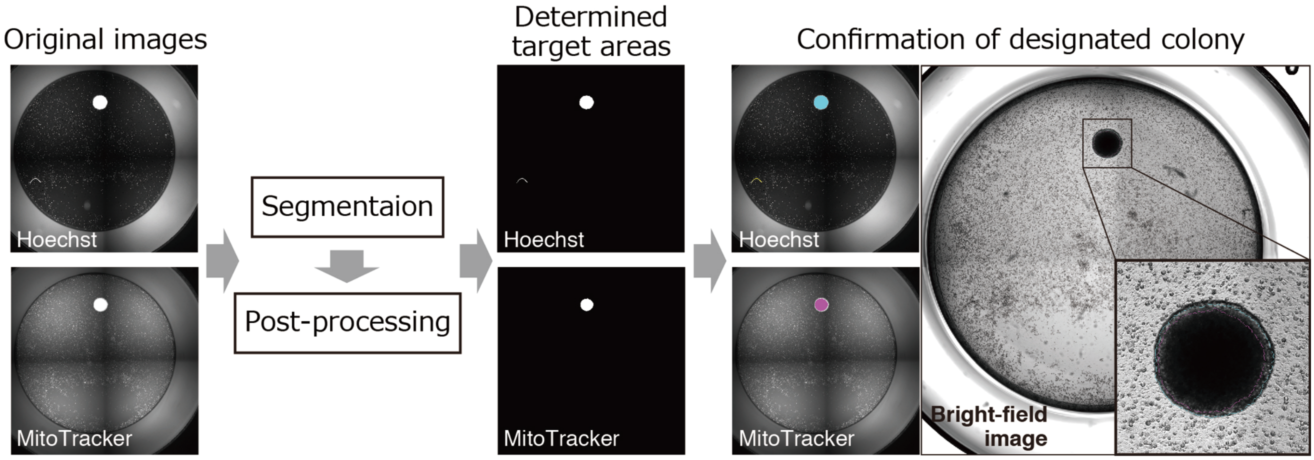

Figure S3. Simple overview of automated image analysis of colonies in soft agar culture

The script for the image analysis merging different colors to detect transformed cell-derived positive colonies was prepared, with the exclusion of false-positive colony images. Initially, the areas showing fluorescent intensity were roughly segmented as an image at each color channel. The recognized areas were refined with a sieve post-processing operation. The criteria of filtering determined the target area at each color channel (size, >6000; circularity, >0.4; fluorescence intensity, >100), and target areas overlapping at two channels were designated as positive colonies. In addition, careful examination of bright-field images was performed to confirm colony formation.

Table S1. Efficiency of single cell-derived colony formation of tumorigenic cell lines in soft agar culture with or without hMSCs (12,500 cells)

	HT-1080	HT-1080/ hMSC	U87	U87/ hMSC	U251	U251/ hMSC
Efficiency of colony formation (%)	15.9 ± 11.1[a]	30.7 ± 14.3	44.9 ± 8.1	64.1 ± 20.3	4.0 ± 1.3	32.6 ± 10.8[#]

[a] The ratio of the total number of colonies to the number of transformed cells dispensed in a 96-well plate were defined as the colony-forming efficiency of transformed cells in soft agar culture. Values are means ± SD of three experiments. Statistical significance was determined using Student's *t*-test ([#] $P < 0.05$, between transformed cells with or without co-cultures). hMSC, human mesenchymal stem cells.
